# Is Sacralization Really a Cause of Low Back Pain?

**DOI:** 10.1155/2013/839013

**Published:** 2013-02-07

**Authors:** Mehmet Bulut, Bekir Yavuz Uçar, Demet Uçar, İbrahim Azboy, Abdullah Demirtaş, Celil Alemdar, Mehmet Gem, Emin Özkul

**Affiliations:** ^1^Department of Orthopaedics and Traumatology, Dicle University Medical School, Diyarbakir, Turkey; ^2^Department of Physical Medicine & Rehabilitation, Dicle University Medical School, Diyarbakir, Turkey

## Abstract

*Objective*. The aim of this study was to determine, by plain radiography, if there is a relationship between sacralization and low back pain. *Methods*. Five hundred lumbosacral radiographs of low back pain patients and 500 control groups were examined. Data collection consisted of the subject's age at the time of imaging, gender, number of lumbar vertebral bodies, and bilateral height measurement of the lowest lumbar transverse process. Dysplastic transverse processes were classified according to the Castellvi radiographic classification system. The incidence of sacralization in patients and the control groups was reported, and the anomaly was compared according to the groups. *Results*. Of these patients groups, 106 were classified as positive for sacralization, which resulted in an incidence of 21.2%. The most common anatomical variant was Castellvi Type IA (6.8%). In the control group, 84 were classified as positive for sacralization, which resulted in an incidence of 16.8%. No statistically significant difference was found between the groups for having sacralization (*P* = 0.09). *Discussion*. The relationship between sacralization and low back pain is not clear. Because of this controversial future studies need to focus on identifying other parameters that are relevant to distinguishing lumbosacral variation, as well as corroborating the results obtained here with data from other samples.

## 1. Introduction

Numerous causes have been attributed to low back pain (LBP). A long list exists, but the enlistment of sacralization as one of the causes has resulted in a lot of controversy. Sacralization is a congenital vertebral anomaly of the lumbosacral spine (fusion between L5 and the first sacral segment) [1]. This alteration may contribute to incorrect identification of a vertebral segment.

Several studies have described the occurrence of this anomaly in a back pain population [2–7]. Some authors have stated that sacralization is incidentally diagnosed and has no clinical impact [7, 8], whereas others claim that this anomaly may predispose patients to certain clinical disorders [9–11]. This controversy has been quite intriguing and has been the stimulus for carrying out this present study. The intention was to examine in detail the incidence of this anomaly in the LBP population. Our study aimed to use the incidence of this congenital anomaly to establish a relationship between it and LBP.

## 2. Materials and Methods

After institution review board approval for this prospective study, 500 lumbosacral radiographs of LBP patients and 500 radiographs of control group were collected over a one-year period. The ages ranged between 16 years and 73 years, and both sexes were involved. Frontal (AP) and lateral lumbosacral regions were evaluated. The radiographs were examined, and data was collected and analyzed. Exclusion criteria consisted of any radiologic evidence of previous lumbosacral surgery that would obstruct our measurements. A total of 1000 lumbosacral films were examined and identified as being adequate for measurement of the desired parameters. Data collection consisted of the subject's age at the time of imaging, gender, number of lumbar vertebral bodies, and bilateral height measurement of the lowest lumbar transverse process. Three orthopedic spine fellows performed all the measurements, using a systemized approach to decrease variability; in addition, consultations between reviewers took place. Digital films were downloaded to an imaging processing program for standardization of the measurements. Subjects without transverse process dysplasia were classified as normal (Type 0), and those with dysplastic transverse process were classified according to the Castellvi radiographic classification system [12] (Table 1). The cases were divided into two groups. Group 1 (G1) included low back pain patients. Group 2 (G2) was the control group. The incidence of sacralizations in the two groups was reported, and the anomaly was compared according to the groups. In addition sacralizations were compared according to gender in the groups.

### 2.1. Statistical Analysis

Statistically significant differences were evaluated by using contingency tables with Fisher's exact test for categorical variables. This test was used to compare statistically the differences between the two groups according to having sacralization. Statistical significance was set at *P* < 0.05.

## 3. Results

In G1, the average age was 39.03 ± 15.9 years (16–73 years). Of these patients (281 women, 219 men), 106 were classified as positive for sacralization, with a gender distribution of 54 (19.22%) women and 52 (23.74%) men. These numbers resulted in an incidence of 21.2% (Table 2). Of the total number of patients (500) seen, 12 (2.4%) had lumbarization. According to sacralization classification, the most common anatomical variant was Castellvi Type IA (6.8%), followed by Type IB (5.4%), Type IIA (1.6%), Type IIB (1.8%), Type IIIA (1.4%), Type IIIB (3.4%), and Type IV (0.8%).

In G2, the average age was 35.4 ± 13.58 years (17–70 years). Of these cases (297 women, 203 men), 84 were classified as positive for sacralization, with a gender distribution of 39 (13.13%) women and 45 (28.48%) men. These numbers resulted in an incidence of 16.8% (Table 3). Of the total number of cases (500) seen, 13 (2.6%) had lumbarization. According to sacralization classification, the most common anatomical variant was Castellvi Type IA (6%), followed by Type IB (4.2%), Type IIA (2%), Type IIB (1%), Type IIIA (0.8%), Type IIIB (1.6%), and Type IV (0.8%).

No statistically significant difference was found between the groups according to the presence of sacralization (*P* = 0.09) (Table 4).

## 4. Discussion

The incidence of sacralization has been reported as 4% to 36% in the general population [1, 6, 9, 13–17]. Its frequency in the low back pain population ranges from 6% to 37% [4, 5, 10, 12, 18–21]. The relationship between LBP and sacralization is not clear. Numerous studies have found no significant correlation between sacralization and low back pain [3, 5, 7, 13, 22], while others have [9–12, 21, 23]. Authors who found no significant correlation [7, 8, 16] have concluded that the incidence of transitional vertebra is equal in those with and without back pain, rendering it only an incidental finding on imaging.

Frymoyer et al. have determined similar rates of radiological abnormalities in three groups of patients: no LBP, moderate LBP, and severe LBP [3]. Similarly, Otani et al. have reported the incidence of sacralization to be 13% in patients with LBP and 11% in their control group [13]. Our study found an incidence rate of 21.2% for sacralization in patients, 16.8% in control group. No statistically significant differences were found between the two groups. Vergauwen et al. demonstrated that the abnormal vertebra does not constitute a risk factor for spine degenerative changes but, when degeneration occurs, it focused on the suprajacent level of the transitional vertebra [22].

Many of the authors with lower estimations used more stringent criteria to count a vertebra as transitional, while other authors did not clearly state all of the inclusion criteria. While Elster [16] and Hsieh et al. [24] reported prevalence as low as 7% and 5.9%, respectively, and Delport et al. [18] observed a rate of 30%, they were based on articulation or fusion of at least one transverse process and presence of an intervertebral disc caudal to the transitional segment, ultimately failing to recognize our most common subtype—Type I. This leaves the current estimates of those with a transitional vertebra in the range of 6%–37% in those seeking healthcare for low back pain, either slightly or significantly higher than the range of 4%–36% commonly reported as the number with sacralization in the general population. This large fluctuation in estimates for the general population amplifies the difficulties in determining the significance of the percentage that we found in our patients. We used the Castellvi radiographic classification system. Castellvi et al. [12] reported a 30% prevalence on his low back pain population. Apazidis et al. [17] found 35.6% prevalence of sacralization in their studies of 211 lumbar spine subjects who had no pain. Their most commonly found pathology was Type IA (14.7%), as in our study.

A great deal of the controversy surrounding the association of sacralization and LBP is the result of an incomplete understanding of the variation present at the lumbosacral junction, as well as the lack of a comprehensive classification scheme that can be used to differentiate lumbosacral variation according to morphological, developmental, and clinical variants. Therefore, a comprehensive understanding of normal and abnormal variations at the lumbosacral junction and a precise classification system are needed to investigate more thoroughly the association between sacralization and LBP. Differences between the classification systems are likely the cause of much of the disagreement; small sample size may also contribute. The controversy in the literature centers on the issue of whether or not sacralization cause low back pain. This confusion may arise from conflicting classifications and descriptions of sacralization. First, an understanding of the variation present must be achieved, complete with a standard, convenient way to categorize that variation. Second, the possible connections among the categories and both frequencies and intensities of low back pain must be investigated as thoroughly as possible.

## Figures and Tables

**Table 1:** Castellvi radiographic classification system of sacralization.

Type IA	A unilateral TP height greater than or equal to 19 mm
Type IB	Both processes height greater than or equal to 19 mm
Type IIA	Presence of unilateral articulation between the TP and the sacrum
Type IIB	Presence of bilateral articulation between the TP and the sacrum
Type IIIA	Unilateral fusion of the TP and the sacrum
Type IIIB	Bilateral fusion of the TP and the sacrum
Type IV	Unilateral Type II transition (articulation) with a Type III (fusion) on the contralateral side

TP: lowest lumbar transverse process.

**Table 2:** Mean age and incidences of the patients in Group 1.

	Mean age (years)	Incidence of sacralization (%)
All patients	39.03 ± 15.9	21.2
Male patients	35.72 ± 15.16	23.74
Female patients	41.60 ± 16.01	19.22

**Table 3:** Mean age and incidences of the cases in Group 2.

	Mean age (years)	Incidence of sacralization (%)
All patients	35.4 ± 13.58	16.8
Male patients	33.5 ± 13.1	28.48
Female patients	36.69 ± 13.77	13.13

**Table 4:** Comparison of the two groups.

	Incidence of sacralization (%)
Group 1	21.2
Group 2	16.8
*P* value	0.09
